# Predicting disease severity in multiple sclerosis using multimodal data and machine learning

**DOI:** 10.1007/s00415-023-12132-z

**Published:** 2023-12-22

**Authors:** Magi Andorra, Ana Freire, Irati Zubizarreta, Nicole Kerlero de Rosbo, Steffan D. Bos, Melanie Rinas, Einar A. Høgestøl, Sigrid A. de Rodez Benavent, Tone Berge, Synne Brune-Ingebretse, Federico Ivaldi, Maria Cellerino, Matteo Pardini, Gemma Vila, Irene Pulido-Valdeolivas, Elena H. Martinez-Lapiscina, Sara Llufriu, Albert Saiz, Yolanda Blanco, Eloy Martinez-Heras, Elisabeth Solana, Priscilla Bäcker-Koduah, Janina Behrens, Joseph Kuchling, Susanna Asseyer, Michael Scheel, Claudia Chien, Hanna Zimmermann, Seyedamirhosein Motamedi, Josef Kauer-Bonin, Alex Brandt, Julio Saez-Rodriguez, Leonidas G. Alexopoulos, Friedemann Paul, Hanne F. Harbo, Hengameh Shams, Jorge Oksenberg, Antonio Uccelli, Ricardo Baeza-Yates, Pablo Villoslada

**Affiliations:** 1 Institut d’Investigacions Biomediques August Pi Sunyer (IDIBAPS) and Hospital Clinic Barcelona, Barcelona, Spain; 2 School of Management, Pompeu Fabra University, Barcelona, Spain; 3 Department of Neurosciences, Rehabilitation, Ophthalmology, Genetics, Maternal and Child Health, University of Genoa, Genoa, Italy; 4 IRCCS Ospedale Policlinico San Martino, Genoa, Italy; 5 University of Oslo, Oslo, Norway; 6 Oslo University Hospital, Oslo, Norway; 7 Institute for Computational Biomedicine, Heidelberg University Hospital, and Heidelberg University, Heidelberg, Germany; 8 Oslo Metropolitan University, Oslo, Norway; 9 Department of Internal Medicine, University of Genoa, Genoa, Italy; 10 Charité Universitaetsmedizin Berlin, Berlin, Germany; 11 Max Delbrueck Center for Molecular Medicine, Berlin, Germany; 12 ProtATonce Ltd, Athens, Greece; 13 School of Mechanical Engineering, National Technical University of Athens, Zografou, Greece; 14 Department of Neurology, University of California, San Francisco, USA; 15 School of Engineering, Pompeu Fabra University, Barcelona, Spain; 16 Department of Medicine and Life Sciences, Pompeu Fabra University, Barcelona, Spain; 17 Hospital del Mar Research Institute, Barcelona, Spain; 18 Present Address: UPF Barcelona School of Management, Balmes 132, 08008 Barcelona, Spain

**Keywords:** Multiple sclerosis, Omics, Imaging, Machine learning, Precision medicine

## Abstract

**Background:**

Multiple sclerosis patients would benefit from machine learning algorithms that integrates clinical, imaging and multimodal biomarkers to define the risk of disease activity.

**Methods:**

We have analysed a prospective multi-centric cohort of 322 MS patients and 98 healthy controls from four MS centres, collecting disability scales at baseline and 2 years later. Imaging data included brain MRI and optical coherence tomography, and omics included genotyping, cytomics and phosphoproteomic data from peripheral blood mononuclear cells. Predictors of clinical outcomes were searched using Random Forest algorithms. Assessment of the algorithm performance was conducted in an independent prospective cohort of 271 MS patients from a single centre.

**Results:**

We found algorithms for predicting confirmed disability accumulation for the different scales, no evidence of disease activity (NEDA), onset of immunotherapy and the escalation from low- to high-efficacy therapy with intermediate to high-accuracy. This accuracy was achieved for most of the predictors using clinical data alone or in combination with imaging data. Still, in some cases, the addition of omics data slightly increased algorithm performance. Accuracies were comparable in both cohorts.

**Conclusion:**

Combining clinical, imaging and omics data with machine learning helps identify MS patients at risk of disability worsening.

**Supplementary Information:**

The online version contains supplementary material available at 10.1007/s00415-023-12132-z.

## Introduction

Developing personalised health care for people with multiple sclerosis (MS) is hindered by our limited understanding of the biological processes underlying the disease, by the lack of validated prognostic or predictive biomarkers and by the clinical heterogeneity between patients [1–4]. At present, clinical decisions are taken based on outcomes identified in natural history cohort studies and randomised clinical trials, such as the disease subtype (relapsing vs. progressive course); age (above ~ 45 years old); the time to reach disability milestones like the expanded disability status scale (EDSS) 4.0 or 6.0; the Evidence of Disease Activity (EDA) [5]; lesion activity (presence of gadolinium-enhancing lesions) and lesion load (presence of new or enlarging T2 lesions and T2 lesion volume) [6]. Indeed, retinal atrophy monitored by optical coherence tomography (OCT) is able to predict the risk of disability worsening [7, 8]. Moreover, the use of disease-modifying drugs (DMDs) and, specifically, high-efficacy therapies, is also associated with a more severe disease course, not the least because they are currently restricted to patients with evidence of a highly active disease [9].

Amongst the biomarkers associated with MS, some have been shown to have a reliable predictive value of a more severe disease course, such as the presence of oligoclonal IgM bands [10, 11], the levels of neurofilaments light [12, 13] or chitinase-3 [14] in the cerebrospinal fluid (CSF) and serum. Although many omic-based biomarkers have been proposed, none has been validated to the level of becoming useful at the individual patient level [2]. Nevertheless, most of these approaches were based on group analysis, which limits their application to individual patients when personalised risk assessment is desired. Accordingly, defining the prognosis of individual patients with MS remains a significant unmet need when considering the application of personalised medicine [3, 15].

In this study, we set out to search for algorithms that stratify MS patients based on a differential risk of disease severity. As such, we combined clinical data with that obtained from neuroimaging and different omics techniques (genomics, cytomics and proteomics) to identify predictors of disease severity [13, 16, 17]. We took advantage of the machine learning tools that tolerate unbalance and overfitting such as random forest algorithms to search in a stepwise manner for the combinations of clinical, imaging and omics variables which identify predictors that are accurate when predicting each clinical outcome [18–22].

## Materials and methods

### Ethical statement

The Sys4MS project was approved by the Institutional Review Boards at each participating institution: Hospital Clinic of the University of Barcelona, IRCCS Ospedale Policlinico San Martino IRCCS, Oslo University Hospital, and Charité—Universitätsmedizin Berlin University. The Barcelona MS cohort study was approved by the Ethical Committee for Clinical Research of the Hospital Clinic Barcelona. Patients were invited to participate by their neurologists, and they provided signed informed consent prior to their enrolment in the study. De-identified data were collected in a REDCap database at the Barcelona centre. All methods were performed in accordance with the relevant guidelines and regulations.

### Patients

The Sys4MS cohort [13, 23] was composed of 322 consecutive MS patients according to 2010 McDonald criteria [24] and 98 healthy controls (HC) at the four academic centres: Hospital Clinic, University of Barcelona, Spain (*n* = 93); Ospedale Policlinico San Martino, Genova, Italy (*n* = 110); Charité University, Berlin, Germany (*n* = 96); and the Oslo University Hospital, Oslo, Norway (*n* = 121). The inclusion criteria were being diagnosed with MS based on 2010 criteria, not having had a relapse in the previous 3 months and patients were required to be stable on the same DMD treatment over the preceding 6 months. RRMS patients were required to have < 10-year disease duration, whereas PMS patients were required to have EDSS 2.0–7.0. The exclusion criteria were use of corticosteroids in the last 30 days, a relapse in the previous 3 months, inability to perform brain MRI, chronic diseases (AIDS, hepatitis B or C, insulin-dependent diabetes, cardiovascular, renal, respiratory or liver insufficiency), pregnancy, breastfeeding or plans to conceive during the course of the study (women only) and participation in any other clinical therapeutic study at or within 30 days of screening visit. We collected clinical information [demographics, relapses, disability scales and use of disease-modifying drugs (DMD)], imaging data (brain MRI and OCT), and blood samples at the same visit. Patients were followed up for 2 years, and the same clinical, disability scales and imaging data (brain MRI and OCT) were collected at the 2-year follow-up visit.

The second cohort was recruited at the Hospital Clinic of Barcelona without overlap with the patients participating in the Sys4MS cohort. The cohort was composed of 271 patients with RRMS or SPMS according to 2010 McDonald criteria [24] and 54 HC without previous or present history of neurological or psychiatric condition. Patients were prospectively recruited at the MS Unit of the Hospital Clinic of Barcelona, as described recently [25].

### Clinical variables

Each patient was assessed on the following disability scales: Expanded Disability Status Scale (EDSS); timed 25 feet walking test (T25WT), 9-hole peg test (9HPT), Symbol Digit Modality Test (SDMT), 2.5% low contrast visual acuity (SL25), and high contrast vision (HCVA, using best corrected acuity, EDTRS charts and logMar transformation) using the conditions indicated in the OCT section. Disability scales were obtained 3 months after any new relapses or use of corticosteroids during the follow-up. We calculated the MS Severity Score (MSSS) and the age-related MS Severity Score (ARMSS) as described elsewhere [26]. No Evidence of Disease activity (NEDA) was defined as no evidence of clinical relapses, new or enlarging T2 lesions and not changes on EDSS [27]. We collected the information regarding the patients’ DMD use, categorised as low-efficacy therapy: interferon-beta, glatiramer acetate and teriflunomide); mid- to high-efficacy therapy: fingolimod, dimethyl-fumarate, natalizumab; or other monoclonals like alemtuzumab, rituximab, daclizumab and ocrelizumab [28]. EDSS and the other clinical scales were confirmed at the end of follow-up based on the results of the 6-month previous clinical visit to define Confirmed Disability Accumulation (CDA). EDSS-based CDA was defined as an increase of one point on the EDSS (for EDSS at baseline between 0 and 5.5) or 0.5 points for patients with EDSS at baseline ≥ 5.5 confirmed at 6 months. For 9HPT, T25WT and SL25, CDA was defined as a 20% change in each score, whereas it was four points for the SDMT confirmed at 6 months [29].

### Imaging

MRI studies were performed on a 3 T scanner at each centre as described before [17], using a standard operating procedure (SOP) to optimise the volumetric analysis. We used the three-dimensional (3D) structural T1-weighted voxel magnetization-prepared rapid gradient echo (T1-MPRAGE) protocol (voxel size: 0.9 × 0.9 × 0.9 mm^3^), with 3D T2-fluid-attenuated inversion recovery (T2-FLAIR) images using the same voxel size to quantify changes in brain volume. Briefly, T2-FLAIR images were registered to T1-MPRAGE scans by a trained technician to ease the manual segmentation of the lesions. Subsequently, lesion in-painting of T1-MPRAGE scans allowed the volume of the whole brain, grey matter and thalamus to be quantified using SIENAX. In addition, we used post-gadolinium T1 axial images (voxel size: 0.7 × 0.6 × 3.0 mm^3^) to quantify gadolinium-enhancing lesions (Gad +). Presence of contrast-enhancing lesions, T2 lesion volume, new or enlarging T2 lesions and volumetric analysis were done at the Berlin centre and by the same operator and were estimated using the lesion in-filled MPRAGE images by FSL SIENAX [30].

Retinal OCT scans were performed in eye-tracking mode by trained technicians under standard ambient light conditions (lighting level of 80–100-foot candles) and without pupillary dilatation, using the same Spectralis device in three centres or a Nidek RS-3000 in Oslo centre. Correction for spherical errors was adjusted prior to each measurement, and the technicians performing OCT scans were blind to the patient’s clinical history. The peri-papillary Retinal Nerve Fibre Layer thickness (pRNFL, μm) was measured with a 12-degree diameter ring scan automatically centred on the optic nerve head (100 ART, 1536 A scans per B scan). The macular scan protocol involved a 20 × 20-degree horizontal raster scan centred on the fovea, including 25 B scans (ART ≥ 9, 512 A scans per B scan). A single grader at Berlin centre at the reading centre in Berlin performed intra-retinal layer segmentation using Orion software^®^ (Voxeleron Inc, Berkeley, US) to quantify the macular ganglion cell plus inner plexiform layer (GCIPL) and the macular inner nuclear layer thicknesses (μm) in the 6 mm ring area as previously described [31]. All OCT scans fulfilled OSCAR-IB criteria [32] and APOSTEL guidelines [33]. Eyes with severe myopia, optic neuropathies or retina diseases were excluded for analysis. We included only eyes without previous optic neuritis (in case both eyes have no previous optic neuritis, the mean of both eyes was used). Scans with an insufficient signal-to-noise ratio, or when the retinal thickness algorithm failed were repeated, or the data were ultimately excluded.

### Genotyping

Genotyping of the samples was performed by Finland Institute of Molecular Medicine Genomics (University of Helsinki, Finland) for the Sys4MS cohort and at the University of California, San Francisco for the Barcelona cohort, using the Illumina HumanOmniExpress-24 v1.2 array (713,599 genotypes from 396 samples). Single-nucleotide polymorphisms (SNPs) imputation was conducted against the 1000-genomes reference (quality of imputation *r*^2^ > 0.5; 6,817,000 genotypes for 396 samples), which allowed us to extract MS-associated SNPs [152 out of 200 known non-HLA MS-associated SNPs available and 17 out of 31 known MS-associated HLA alleles available (HLA*IMP programme)] as described elsewhere [34]. The MS Genetic Burden Score (MSGB) is used as cumulative genetic risk estimations for MS patients. The MSGB for the HLA and non-HLA alleles and their combination were calculated as described previously [35]. Briefly, the MSGB is computed based on a weighted scoring algorithm using one SNP per MS-associated genomic region as found by trend-test association (meta-) analysis. This statistic is an extension of the log additive model, termed “Clinical Genetic Score”, with weights given to each SNP based on its effect size as reported in the literature. The MSGB is obtained by summing the number of independently associated MS risk alleles weighted by their beta coefficients, obtained from a large GWAS meta-analysis, at 177 (of 200) non-MHC (major histocompatibility complex) loci and 18 (of 32) MHC variants, which includes the HLA-DRB1*15:01-tagging single-nucleotide polymorphism (SNP) rs3135388 [36–40].

### Cytomics

Cytomics was performed on fresh peripheral blood mononuclear cells (PBMCs) using 17 antibodies that covered 11 subpopulations of T, B and NK cells as described in detail elsewhere [23]. The following cell populations were studied: Effector cells: Th1 classic: CD3 + CD4 + CxCR3 + CCR6-CD161−; Th17: CD3 + CD4 + CxCR3 + CCR6-CD161 + CCR4 +; Th 1/17: CD3 + CD4 + CCR6-CD161 + CxCR3highCCR4low; Regulatory T cells: CD3 + CD4 +: T reg CD25 + CD127-, T naive CD45RA + CD25low; CD3 + CD8 +: T reg CD28− and T naive CD28-CD45RA +; B cells: B memory: CD19 + CD14-CD24 + CD38−; B mature: CD19 + CD14-CD24 + CD38low; B regulatory: CD19 + CD24highCD38high and NK cells: Effector: CD3-CD14-CD56dim: Regulatory: CD3-CD-CD56bright (reg).

### Phosphoproteomics

The phosphorylation levels of 25 kinases participating in pathways associated with MS [41] (AKT1, AKTS1, CREB1, GSK3AB, HSPB1, IKBA, JUN, KS6B1, LCK, MK12, MK03/01, MK09, MP2K1, NRF2, P53, PGFRB, PTN11, RS6, SRC, STAT1, STAT3, STAT5, STAT6, TF65, WNK1) were assessed by xMAP assays in PBMCs and quantified as previously described [42].

### Machine learning analysis

The search for predictors of clinical outcomes (see the list of outcomes on supplementary file) was performed through a machine learning analysis using Python and the Scikit Learn library (scikit-learn.org). The analysis included 100 features: clinical, demographics, disability scales, DMD use, MRI, OCT, MSGB, cytomics and phosphoproteomics (supplementary file). Initially, we calculated the Pearson correlation matrix for the different groups of variables (clinical, MRI, OCT, MSGB, cytomics and phosphoproteomics) to select the most informative features based on showing correlation >|0.6| to exclude co-linear variables. In this way, multidimensionality was balanced between the number of features and the number of samples, maintaining a ratio of 1:5 [43]. Besides, we explore further reducing dimensionality using principal component analysis on the features selected. The search of classifiers was done using Random Forest algorithms, considering they are better in handling unbalanced data, high dimensionality, multi-collinear features and have a lower risk of overfitting, which is a common problem in biomedical datasets [44], when studying complex disorders such as MS [20]. For a classification of the clinical endpoints, we calculated the entropy, defined as the measure of impurity, following the formula:

$${\text{Entropy}} = \sum \limits_j p_j \log_2 p_j$$

where $p_j$ is the probability of class *j.*

During training, several random forest parameters were automatically optimised based on the: (1) number of estimators (number of trees in a random forest): the best value among 10 equally spaced values between number_features/4 and number_features/2; (2) maximum depth (levels in the tree); (3) minimum number of samples required to split a node; and (4) minimum number of samples needed for each leaf node: the best value amongst [19, 44].

We conducted a feature selection process using the *feature importance* algorithm [44] for selecting the most informative variables and, in this way, increase accuracy, reduce overfitting and reduce training time. Feature importance was calculated as the decrease in node impurity (Gini index) weighted by the probability of reaching that node as defined in the following formula

$$\sum_{i = 1}^C {f_i \left( {1 - f_i } \right)}$$

where *f*_*i*_ is the frequency of label *i* at a node, and *C* is the number of unique labels.

The node probability was calculated by the number of samples that reach the node, divided by the total number of samples; therefore, the higher the value, the more critical the feature*.*

Unbalanced data were addressed by applying cost-sensitive learning, wherein classes were automatically weighted inversely proportional to how frequently they appear in the data [43]. Missing data were addressed as follows: (1) by removing features with more than 20% of missing data when studying the effect of features different from the previous ones; (2) by eliminating observations (patients) with missing data in features with more than 20% of missing data, when studying the effect of these features; and (3) for the remaining missing data, we build a regression model using the random forest for each variable with tenfold cross-validation with all data, and we used a random grid to search for hyper-parametrization. Regarding the dynamic programming problem of “curse of dimensionality”, we applied the rule that there should be at least five training examples for each dimension in the representation (the minimum for each category should be at least 5 cases). For these classification problems, we calculated balanced recall (sensitivity), precision (positive predictive value), and F1 (harmonic mean of recall and precision) measures. The area under the receiver operating curve (AUC) was calculated for the predictors with accuracy above 70%.

## Results

### The Sys4MS cohort

We recruited 322 consecutive MS patients (age 41 ± 10 years, 71% female), of which 271 (82%) had Relapsing–Remitting MS (RRMS), and 57 (18%) had Progressive MS (PMS; 28 had SPMS and 29 had PPMS), as well as 98 healthy controls matched by sex and age with the RRMS group (Table 1). The patients had a mean disease duration of 10 years, a median EDSS of 2.0 (range 0–8), and mean MSSS of 3.6. Regarding the use of therapies at baseline, 70% of patients were being treated with DMDs, 44% with low-efficacy therapies and 26% with high-efficacy therapies. Clinical and imaging (MRI and OCT) characteristics of the subjects at baseline are summarised in Table 1 and supplementary file S1.

**Table 1:** The Sys4MS cohort: clinical and imaging variables at baseline

	MS baseline	MS 2-year FU	HC
	*n* = 322	*n* = 278	*n* = 98
Age	41 (10)	45 (9.8)	36.98 (11.4)
Female	229 (71%)	194 (70%)	63 (70%)
Age at disease onset (years)	31 (9)	31 (9)	–
Disease duration (years)	10 (8)	12.9 (8.16)	–
Subtype			
RRMS	271	228	
SPMS	28	25	–
PPMS	29	25	
EDSS	2.0 (0–8.0)	2.0 (0–8.0)	–
MSSS	3.6 (2.2)	3.25 (2.35)	–
ARMS	3.9 (2.1)	3.56 (2.26)	–
T25WT (sec)	6.93 (6.6)	5.67 (4.97)	–
9HPT (sec)	21.2 (6.5)	21.9 (5.92)	–
SDMT (# symbols)	53.8 (13.5)	53.5 (13.3)	–
SL25 (# letters)	29.1 (13.4)	26.7 (13.5)	–
HCVA (LogMAR)	0.03 (0.36)	-0.11 (0.44)	–
DMD			
Untreated	91	72	–
Interferon beta	43	19	–
Glatiramer acetate	39	24	–
Teriflunomide	28	21	–
Fingolimod	38	33	–
Dimethyl-Fumarate	35	37	–
Natalizumab	29	24	–
Other	19^a^	43^b^	–

MRI			
# Gadolinium lesions	0.1 (0.5)	NA	NA
T2 lesion volume (cm^3^)	8.17 (10.5)	9.32 (11)	NA
NBV (cm^3^)	1509 (91)	1454 (70.2)	1587 (58.9)
NGMV (cm^3^)	792 (65)	779 (49.5)	856 (48.3)
NWMV (cm^3^)	716 (68)	676 (43.5)	731 (31.8)

OCT (µm)	OD	OS	
pRNFL (µm)	100 (12.7)	101 (12.1)	NA
mRNFL (µm)	39.6 (4.9)	39.6 (4.31)	–NA
GCIPL (µm)	65.6 (8.3)	65.7 (7.08)	–NA
INL (µm)	31.5 (2.8)	31.5 (2.77)	–NA
ORL (µm)	146.1 (9.5)	147 (8.39)	–NA

Disability scales are shown as the mean (standard deviation or range), except for the EDSS which is displayed as the median (range) and gender which is shown as the n and percentage

NA, not available

^a^Other DMD baseline: alemtuzumab: 9, rituximab: 7, ocrelizumab: 1, daclizumab: 2

^b^Other DMD year 2: alemtuzumab: 13, rituximab: 11, ocrelizumab: 16, cladribine: 3

By the end of follow-up (mean follow-up 1.98 ± 0.94 years, *n* = 274), 2 RRMS cases had progressed to SPMS, 22 patients had started DMDs (Cladribine: 1; Fingolimod: 2; Glatiramer acetate: 4; Ocrelizumab: 9; Rituximab: 2; Teriflunomide: (4) and 17 had changed from low to high-efficacy therapies. The number of cases with confirmed disability accumulation (events) for each of the scales was as follows: EDSS: 52, T25WT: 30, 9HPT: 11, SDMT: 27 and SL25: 75; and 122 patients remained as NEDA. Table 1 summarises the frequency of each therapy and means disability scales at the follow-up visit.

### Omics analysis

From the HumanOmniExpress-24 v1.2 array, we imputed 152 SNPs outside the HLA region associated with and17 HLA-class II alleles. The MSGB, only the HLA alleles genetic burden score (MSGB^HLA^), and the non-HLA genetic burden score (MSGB^non-HLA^) were calculated. As expected, the MSGB was significantly higher in the MS patients than in the HC group for the MSGB (MS = 4.23 and HC = 3.2; *p* = 3.4 × 10^–8^); MSGB^HLA^ (MS = 1.57 and HC = 0.95; *p* = 1.6 × 10^–4^); and MSGB^non-HLA^ (MS = 2.6 and HC = 2.2; *p* = 6.8 × 10^–5^). Of the 322 patients and 98 HCs recruited, a flow cytometry analysis was carried out on the first 227 consecutive patients and 82 HCs, which did not differ from the overall cohort in the baseline characteristics. Results of the cytomics analysis in this cohort are described in detail elsewhere [23]. Briefly, significantly higher frequencies of Th17 cells in the RRMS population compared with HC and lower frequencies of B memory/B regulatory cells as well as higher percentages of B mature cells in patients with PMS compared with HCs were found. In addition, we observed higher percentages of B mature cells in patients with PMS compared with HCs. Fingolimod treatment induced a decrease in total CD4 + T cells and in B mature and B memory cells and increases in CD4 + and CD8 + T regulatory and B regulatory cells [23]. Finally, the phosphoproteomic analysis was carried out on the first 148 consecutive MS patients, which did not differ from the overall cohort in the baseline characteristics. Patients showed higher levels of phosphorylated IKBA, JUN, KSGB1, MK03, RS6, STAT3 and STAT6 in MS patients compared to controls. See supplementary file for aggregated results for each variable.

### Predictors of disease activity

We searched for algorithms predicting clinical outcomes at follow-up, such as 6-month confirmed disability accumulation using the EDSS, T25WT, 9HPT, SDMT or SL25 scales, as well as maintaining the NEDA status or starting or changing DMDs, using random forests algorithms (see supplementary file for list of outcomes and features). We compared algorithm performance based on the use of clinical data alone, or by adding imaging, genetics, and the other omics information sequentially to learn how much the prediction improves by including additional tests. This stepwise approach was chosen for prioritising algorithms based on the accuracy but also the consequent burden for patients and health systems depending on the tests required (genetics was analysed separately form the other omics because the accessibility of genotyping at present). We found algorithms with AUC higher than 60% for most of the outcomes, and AUC above 80% for SL25 and NEDA status during follow-up (Table 2). We tested also the performance of using support vector machines without finding improvement on the accuracy of the classifiers (data not shown).

**Table 2:** Algorithm performance for predicting clinical outcomes at 2-year follow-up for the Sys4MS cohort

Outcome	Features	# Patients	Precision	Recall	F1	Accuracy*	AUC
EDSS (delta)	C	262	0.63	0.63	0.63	0.58	0.56
	**C/I**	262	0.64	0.65	0.64	0.58	0.57
	C/I/G	262	0.60	0.64	0.61	0.57	0.54
	C/I/O	84	0.71	0.61	0.63	0.54	0.41
EDSS (CDA)	C	262	0.71	0.73	0.72	0.52	0.62
	C/I	262	0.68	0.71	0.69	0.47	0.57
	C/I/G	262	0.69	0.70	0.69	0.52	0.59
	**C/I/O**	84	0.85	0.85	0.85	0.62	0.45
9HPT (CDA)	C	228	0.90	0.93	0.92	0.49	0.61
	**C/I**	228	0.90	0.93	0.92	0.48	0.65
	C/I/G	228	0.90	0.93	0.92	0.49	0.61
	C/I/O	71	0.81	0.90	0.85	0.50	0.65
T25WT (CDA)	C	224	0.75	0.80	0.78	0.50	0.44
	**C/I**	224	0.75	0.80	0.77	0.45	0.50
	C/I/G	224	0.75	0.80	0.77	0.46	0.48
	C/I/O	68	0.85	0.85	0.85	0.46	0.45
SDMT (CDA)	C	238	0.83	0.87	0.85	0.49	0.63
	C/I	238	0.81	0.84	0.83	0.48	0.62
	**C/I/G**	238	0.81	0.83	0.82	0.47	0.61
	C/I/O	73	0.75	0.73	0.74	0.51	0.40
SL25 (CDA)	**C**	212	0.82	0.82	0.82	0.63	0.81
	C/I	212	0.82	0.82	0.82	0.61	0.80
	C/I/G	212	0.81	0.81	0.81	0.62	0.79
	C/I/O	63	0.76	0.84	0.80	0.48	0.47
NEDA	C	146	0.75	0.75	0.75	0.58	0.80
	C/I	146	0.76	0.76	0.76	0.59	0.80
	**C/I/G**	146	0.76	0.76	0.77	0.60	0.79
	C/I/O	46	0.83	0.91	0.87	0.50	0.68
Change to high efficacy	C	275	0.89	0.94	0.91	0.49	0.68
	**C/I**	275	0.89	0.93	0.91	0.48	0.64
	C/I/G	275	0.89	0.93	0.91	0.50	0.54
	C/I/O	89	0.87	0.92	0.89	0.49	0.12
Starting therapy	C	142	0.74	0.76	0.75	0.60	0.65
	C/I	142	0.70	0.71	0.71	0.52	0.66
	C/I/G	142	0.68	0.70	0.69	0.50	0.54
	**C/I/O**	38	0.80	0.82	0.80	0.69	0.50

Outcomes included the change on the EDSS, confirmed disability accumulation (CDA) of disability scales, remaining in No Evidence of Disease Activity (NEDA) by end of follow-up, change to high-efficacy drugs and starting disease-modifying therapies during follow-up. Features tested are shown by the type of variables [clinical (C), imaging (I), Genetics (G) and omics (O)]. Results are shown as precision (positive predictive value), recall (sensitivity), F1 (harmonic mean of precision and recall), the *balanced accuracy, and area under the receiver operator curve (AUC)

CDA, confirmed disability accumulation; EDSS, Expanded disability status scale; 9HPT, nine-hole peg test; T25WT, timed 25-feet walking test; SDMT, symbol digit modality test; SL25, Sloan low-contrast visual acuity 2.5%; NEDA, no evidence of disease activity; DMD, disease-modifying drug

**Fig. 1:**
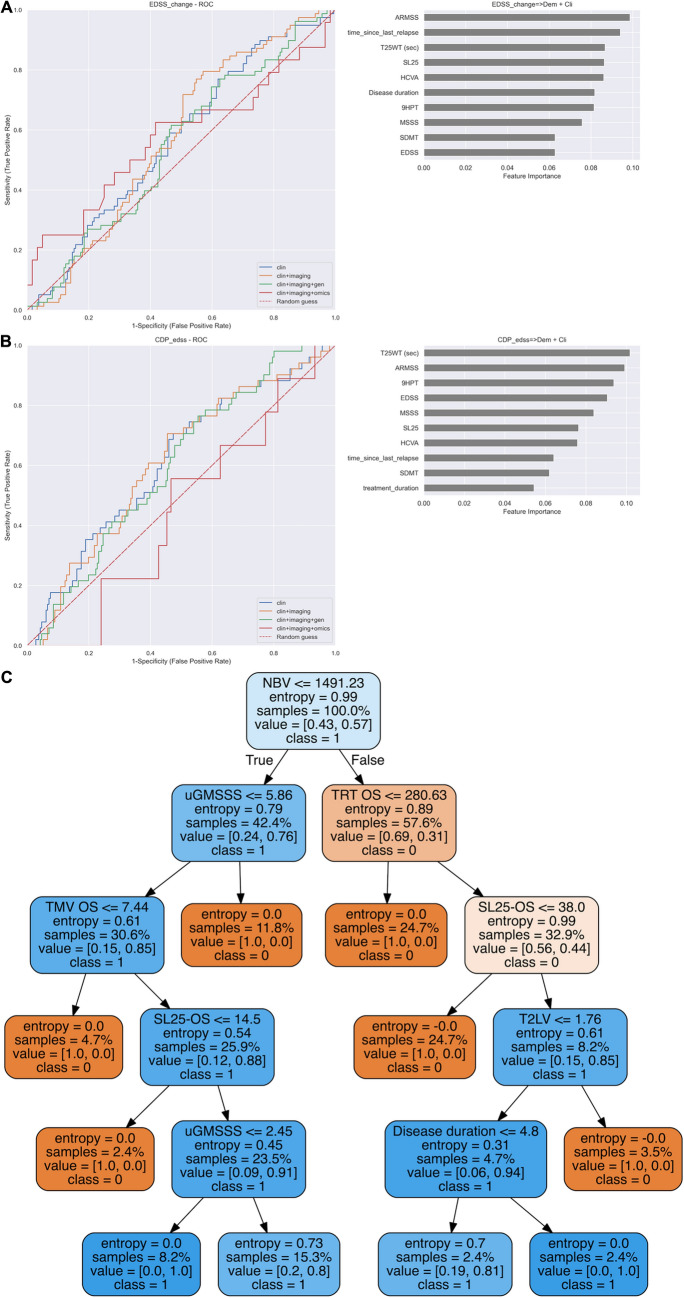
Performance of random forest algorithm for predicting the EDSS at 2-year follow-up. **A** ROC curve showing 6-month confirmed EDSS accumulation at the end of follow-up using: (1) clinical features (blue); (2) clinical and imaging (MRI and OCT) features (orange); or (3) clinical, imaging and omics features (green); the random classification is shown in red. **B** Representative tree of the random forest for predicting 6-month confirmed EDSS accumulation. Each box of the decision trees shows the following information: (1) feature of the tree: based on the result, it either follows the true or the false path; (2) entropy, a measure of disorder or uncertainty that is reduced by the algorithm; (3) samples: percentage of samples that fall in that node; (4) value: the proportion of samples that falls in each category (class); and (5) class. **C** discriminatory features by order of relevance (top to down) for the best predictors of EDSS-CDA based on the AUC using: **a** clinical, **b** clinical and imaging, **c** clinical, imaging and genetics, and **d** Clinical, imaging, genetics and omics features

At present, the gold standard for defining disease progression in patients with MS is by probing 3- or 6-month confirmed disability accumulation of the EDSS (EDSS-CDA) [29]. We found random forest algorithms predicting which MS patients would achieve EDSS-CDA by 6-month on the EDSS 2 years later with precision (positive predictive value): 71%, recall (sensitivity): 73%, and F1 (harmonic mean): 72% (Table 2 and Fig. 1a, b). The accuracy of the predictor did not increase from using clinical features alone to adding imaging, genetic or omics information (a representative decision tree of including clinical and imaging features is shown in Fig. 1c). The predictors always included disability scales, age or disease duration as the most informative features, followed by brain volume and T2 lesion load, whereas the only omics that contributed to predictors was phosphoproteomics, including the levels of phosphorylated MP2K1, a kinase of the MAPKinase pathway associated with MS [42].

**Fig. 2:**
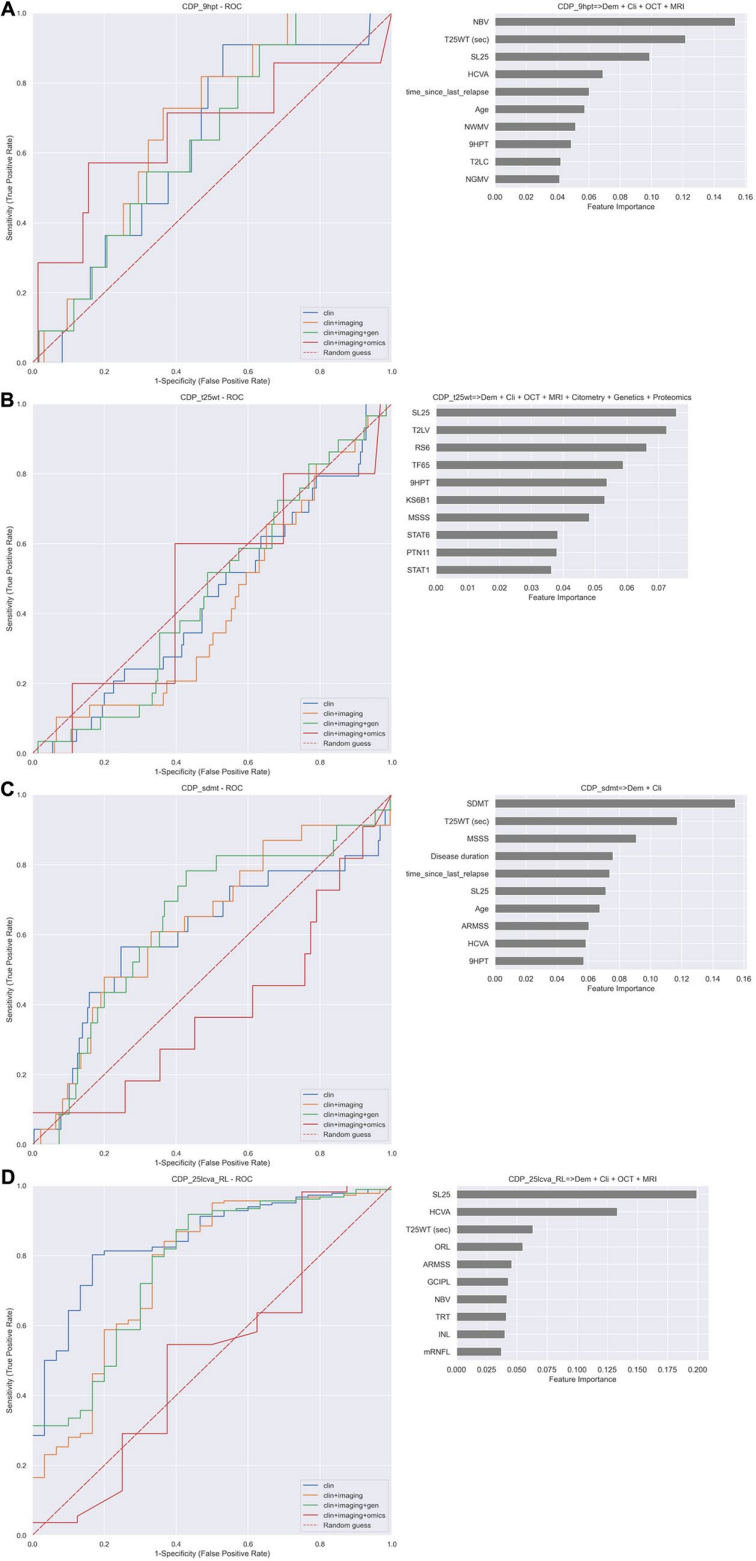
Performance of random forest algorithm for predicting confirmed disability accumulation disability scales at 2-year follow-up. The figure shows the ROC curves on the left and the feature importance ranking (top 10) for the best predictor (Table 2) on the right. **A** 9HPT; **B** T25WT; **C** SDMT; **D** SL25

Regarding the prediction of confirmed disability accumulation for other disability scales, the algorithm for predicting 9HPT using clinical and imaging features achieved a precision 90%, recall 93% and F1 92%, with disability scales, brain volume and T2 lesion volume (T2LV) being the most informative (Fig. 2a). The algorithm for predicting the T25WT achieved a precision 75%, recall 80% and F1 77%, by combining clinical, imaging and omics data, with several kinase phosphorylation levels (JUN, STAT6, MP2K1, AKT1, PTN1 and GSK3B), disease duration and disability scales being the top predictors (Fig. 2b). The algorithm for predicting SDMT also achieved a precision 83%, recall 87% and F1 85%, when using clinical and imaging variables, with disability scales, brain volume and T2LV being the most informative ones (Fig. 2c). Moreover, the algorithms for predicting the SL25 achieved a precision 82%, recall 82%, and F1 82%. In this case, the informative variables were visual acuity at baseline, retina thickness (pRNFL), and several kinase levels (Fig. 2d).

**Fig. 3:**
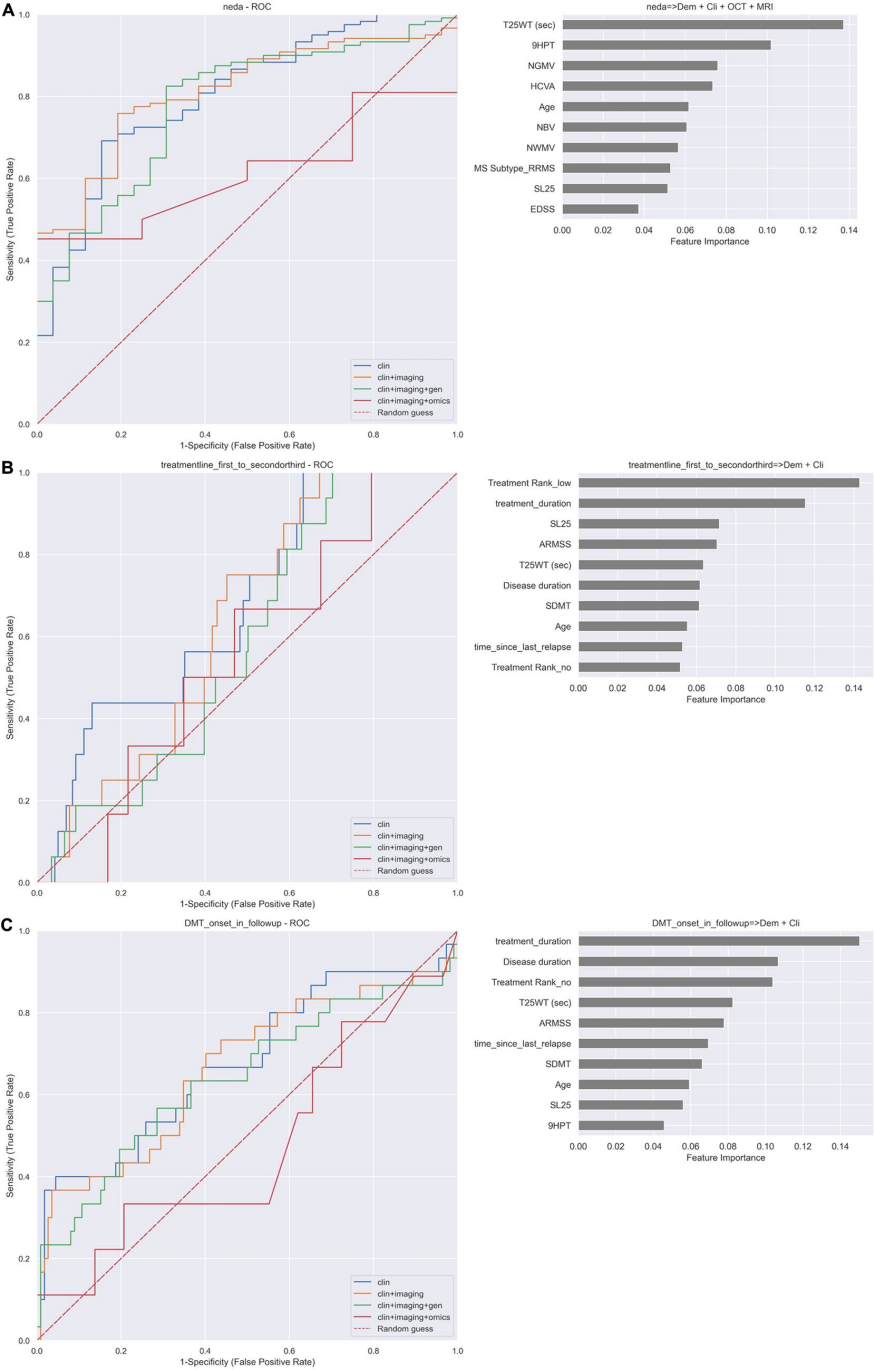
Performance of random forest algorithm for predicting NEDA and change on therapy during follow-up. The figure shows the ROC curves on the left and the feature importance ranking (top 10) for the best predictor (Table 2) on the right. **A** staying on NEDA after 2 years follow-up; **B** starting DMDs during follow-up; **C** change from the first line to high-efficacy DMDs during follow-up

We also searched for algorithms predicting maintaining the NEDA status by the end of follow-up, obtaining a precision 76%, recall 76%, and F1 76%. The algorithm for predicting NEDA included as top features several disability scales, age, disease duration and disease subtype (Fig. 3a).

### Predictors of the use of disease-modifying drugs

At present, therapeutic decisions are based on the label of approved drugs, but personalization of the therapeutic recommendation is a sought for goal. We analysed whether MS patients changing from the low- to high-efficacy therapies or who started DMD during the 2-year follow-up period. We found a random forest algorithm for the change from the low- to high-efficacy therapies using only clinical variables, with precision 89%, recall 94% and F1 91%, and such performance did not improve by adding imaging, genetics or other omics (Table 2). The most informative variables for the change to high-efficacy therapies were the treatment line at baseline, treatment duration, disease duration, time since last relapse, age and disability scales (Fig. 3b). Indeed, we searched for algorithms predicting the onset of new DMD for treatment-naïve MS patients with precision 70%, recall 71% and F1 71% by including only clinical data (Table 2). The most informative variables were treatment duration and high- versus low-efficacy therapy, disease duration, time since the last relapse, age and disability scales (Fig. 3c).

### Assessment of algorithm accuracy in the Barcelona cohort

In order to test the accuracy of the predicting algorithms, we repeated the analysis in an independent prospective cohort from a single centre that includes clinical, imaging and genomic features similar to that of the Sys4MS cohort (cytomics and phosphoproteomics data were not available from this cohort). The cohort was composed of 271 patients with RRMS or SPMS and 54 HC (Table 3).

**Table 3:** The Barcelona cohort: clinical and imaging variables at baseline

	MS baseline	MS 2-year FU	HC
	*n* = 251	*n* = 235	*n* = 24
Age	43.5 (10.7)	44.8 (10.8)	39.4 (10.2)
Female	180 (72%)	169 (72)	19 (79%)
Age at disease onset (years)	31.4 (25.7–38.9)	31.1 (25.5–38.9)	–
Disease duration (years)	9.9 (2.4–15.7)	10.3 (3.6–17)	–
Subtype			
CIS	22 (9)	4 (2)	
RRMS	203 (81)	183 (887)	
SPMS	20 (8)	16 (8)	
PPMS	6 (2)	6 (3)	–
EDSS	2.0 (0–6.5)	2.0 (0–7.0)	–
MSSS	2.9 (1.8–4.9)	2.7 (1.7–4.2)	–
ARMS	3.2 (2.1–4.9)	3 (1.9–4.6)	–
T25WT (sec)	4.2 (3.8–5.2)	4.5 (3.9–5.6)	–
9HPT (sec)	20.8 (19–23.5)	21 (18.7–24)	–
SDMT (# symbols)	50.1, 13.2	51.6, 13.2	–
SL25 (# letters)	27 (16.2–30.5)	22.5 (13–30)	–
HCVA (LogMAR)	0 (-0.1–0)	0 (-0.1, 0.1)	–
DMD			
Untreated	122 (48)	23 (26)	–
Interferon beta	67 (27)	35 (39)	–
Glatiramer acetate	28 (11)	13 (15)	–
Teriflunomide	8 (3)	1 (1)	–
Fingolimod	9 (4)	9 (10)	–
Dimethyl-Fumarate	3 (1)	2 (2)	–
Natalizumab	12 (5)	4 (5)	–
Other	2 (1)^a^	2 (2)^b^	–

MRI			
T2 lesion volume (cm^3^)	5.1 (2.2–11.4)	2.1 (0–7.8)	–
NBV (cm^3^)	1505 (124)	1405 (97.5)	–
NGMV (cm^3^)	722.4 (65.3)	662.6 (48.5)	–
NWMV (cm^3^)	778.1 (66.7)	742.4 (63.3)	–

OCT (µm)			
pRNFL (µm)	90.5 (80.5–100.2)	88.2 (79.4–97.6)	–
mRNFL (µm)	26.4 (23.7–28.4)	25.9 (23.6–28.4)	–
GCIPL (µm)	68.2 (61.8–74.5)	67.1 (59.9–73.5)	–
INL (µm)	37.2 (34.9–39.1)	37.1 (35.1–39.1)	–
ORL (µm)	110.1 (106.1–113.9)	109.6 (105.5–113.1)	–

Disability scales are shown as the mean and standard deviation or range, except for the EDSS which is displayed as the median (range)

^a^Other DMD baseline: alemtuzumab: 9, rituximab: 7, ocrelizumab: 1, daclizumab: 2

^b^Other DMD year 2: alemtuzumab: 13, rituximab: 11, ocrelizumab: 16, cladribine: 3

The analysis was conducted by training the random forest algorithm with the new data, considering that differences in the calculation of several clinical, imaging and MSGB variables would prevent the direct use of the trained algorithm. We found comparable accuracy for the confirmed EDSS worsening as well as for most of the outcomes (Table 4). For example, the change on EDSS, 9HPT, T25WT and SDMT CDA achieved similar AUC, but it was slightly higher (from 62 to 77%) for the EDSS-CDA and smaller for the SL25 CDA (from 81 to 67%) in the Barcelona cohort.

**Table 4:** Random forest algorithm performance for predicting clinical outcomes at 2-year follow-up

Outcome	Features	Precision	Recall	F1	Accuracy*	AUC
EDSS (delta)	C	0.60	0.58	0.59	0.5360	0.60
	C/I	0.62	0.60	0.61	0.566	0.56
	C/I/G	0.64	0.61	0.62	0.564	0.54
EDSS (CDA)	C	0.86	0.86	0.86	0.6777	0.77
	C/I	0.81	0.78	0.79	0.5775	0.75
	C/I/G	0.83	0.79	0.81	0.5776	0.76
9HPT (CDA)	C	0.83	0.89	0.86	0.4965	0.65
	C/I	0.83	0.88	0.86	0.4863	0.63
	C/I/G	0.83	0.88	0.86	0.4864	0.64
T25WT (CDA)	C	0.67	0.69	0.68	0.564	0.54
	C/I	0.69	0.62	0.64	0.6148	0.48
	C/I/G	0.69	0.62	0.64	0.6148	0.48
SDMT (CDA)	C	0.95	0.96	0.95	0.686	0.66
	C/I	0.91	0.95	0.93	0.4965	0.65
	C/I/G	0.91	0.95	0.93	0.4965	0.65
SL25 (CDA)	C	0.62	0.62	0.62	0.627	0.67
	C/I	0.63	0.62	0.62	0.634	0.64
	C/I/G	0.66	0.64	0.62	0.638	0.68

Features tested are shown by the type of variables (clinical (C), imaging (I) and genetics (G) and the number of features used. Results are shown as precision (positive predictive value), recall (sensitivity), F1 (harmonic mean of precision and recall), the *balanced accuracy and the area under the receiver operator curve (AUC)

CDA, confirmed disability accumulation; EDSS, Expanded disability status scale; 9HPT, nine-hole peg test; T25WT, timed 25-feet walking test; SDMT, symbol digit modality test; SL25, Sloan low-contrast visual acuity 2.5%; NEDA, no evidence of disease activity; DMD, disease-modifying drug

## Discussion

In this study, we searched for predictors of future disease activity in MS by combining longitudinal clinical and imaging, with omics information, and applying machine learning algorithms such as random forest. We were interested in identifying predictors for each of the outcomes, as well as establishing the contribution of each type of variable (clinical, imaging, omics) to the predictors to assess the feasibility of the algorithms in clinical practice. We found predictors with mid- to high-accuracy for several disability outcomes, such as confirmed disability progression on the EDSS, 9HPT, SDMT and SL25. The main variables contributing to such predictors were always disability scales at baseline, followed by brain or retina atrophy variables, and proteomics variables. Such level of accuracy was assessed in a second and independent cohort.

Recent studies have addressed the ability of brain MRI to predict the course of MS using deep learning, finding good accuracy for predicting clinical worsening [45]. Regarding the use of DMD as a surrogate marker of disease activity, we analysed the ability to predict the start of the DMD or the switch to high-efficacy therapies, two relevant milestones in MS care. It is well described that disease activity and age are strong predictors of response to therapy [46], but also differences in cell populations, such as B (CD19 + CD5 +) and CD8 (perforin +) T cells, are associated with a differential response to some therapies, such as INFB [47], natalizumab or fingolimod [23]. Indeed, the recently developed Individual Treatment Response (ITR) score for MS therapies also identified clinical disability, quality of life and some imaging outcomes as the main predictors of response to therapy [48]. Our machine learning study identified algorithms with high accuracy for predicting the escalation of therapy from the first-line to high-efficacy DMD.

An in-depth analysis of molecular changes by omics analysis offers the promise of providing a comprehensive picture of the pathways altered in complex diseases and consequently improve our prediction of the course of the disease [49, 50]. In the case of MS, other omics approaches have been tested for predicting disease prognosis or response to therapy including pharmacogenetics [51, 52], gene expression [53], proteomics [21, 53], metabolomics [54, 55] or phosphoproteomics [42] analysis aimed to interrogate signalling pathways driving tissue damage and clinical phenotype [2, 41]. By examining signalling pathways by phosphoproteomics and making use of systems biology modelling, it has been possible to identify signalling networks associated with the use of MS therapies at the individual patient level [56]. However, most of such approaches have not achieved very high accuracy and has not been validated to be of use in clinical practice [2]. For this reason, validation of the biomarkers identified so far, combined with prospective multicentric studies, will be required for generating the evidence to be applied in personalised medicine.

In this study, we have applied random forest algorithms for searching the combination of variables that better explain the outcome 2 years later because they better tolerate data unbalance and overfitting. Random forest allows developing algorithms for classification (dichotomous outcomes) or regression (continuous outcomes) by constructing decision trees, ranking variables by importance, and without overfitting the training set. For these reasons, they are being applied to omics and imaging classification problems [18, 19] and are the most commonly used in MS [20–22]. Other machine learning techniques can be applied to this type of datasets, such as neural networks, linear regression or least absolute shrinkage and selection operator (LASSO) regression methods, support vector machines or Bayesian networks, which may differ in their performance depending on the size of the dataset and quality of the data as well as on the type of prediction or clinical question [16, 57–60]. However, the main limitation, in addition to the sample size, is having variables sensitive to the outcome to be predicted [61]. Indeed, we tested support vector machines in this dataset without achieving higher accuracies compared to random forest algorithms. Informative variables are quite difficult to obtain in brain diseases because current assessments may not be sensitive to minor changes in the evolution of the illness, due to the lack of specificity for the biological substrate or lack of spatial and temporal resolution. Whilst machine learning can be effectively used to model well-defined systems, its application to complex diseases dictates a much more careful approach, including high-quality data, expert knowledge and significant customization to the specific medical question being addressed. Finally, differences between centres in terms of patient population, use of DMD or methods for collecting and calculating clinical or imaging variables are other sources of noise for this type of analysis, even if we made significant efforts to standardised data collection between centres.

Physicians would benefit for their natural Bayesian thinking by updating the prior probabilities (e.g. risk of progression or response to therapy based on clinical judgement) with the likelihood ratios (based in the sensitivity and specificity of the biomarkers) obtained from clinical monitoring, imaging or omics to improve their predictions (posterior probabilities) [62]. One formal application already available for MS patients management is the Bayesian Risk Estimate for MS (BREMS) [63], which updates the prior probabilities based on age and disability scales (EDSS) for predicting the MSSS and the conversion to SPMS [64]. Further refinement of these algorithms based on decision trees or Bayesian networks would help support the reasoning and decision-making process for the management of care for people with MS.

The main limitation of the study is the limited sample size considering the heterogeneity, noise and missing data for the machine learning approach. Although we collected a prospective multicentric cohort of more than 300 cases with a 2-year follow-up with a comprehensive assessment with clinical information, disability scales, quantitative imaging and omics information, the sample size was far from being big data, and a follow-up of 2 years is limited to identify enough events for the outcome variables. In addition, some patients dropped out, or some assessment was not completed, creating data gaps that impaired the algorithm performance. Our study did not include relevant CSF-based biomarkers such as IgM oligoclonal bands or chitinase because lack of CSF samples and to avoid requesting a lumbar tap as inclusion criteria to facilitate recruitment. More, spinal cord MRI were also not collected, missing the presence of spinal cord lesions as a predictor. Finally, due to the differences in how some features were calculated between both cohorts (e.g. different method for the imaging analysis and MSGB calculations), this prevented to validate the algorithm in the second cohort. Indeed, the study includes imaging biomarkers but not molecular biomarkers such as oligoclonal bands or neurofilaments that may have improved the algorithm performance. However, even with such limitations, we were able to identify algorithms with fair to good accuracy for predicting relevant clinical outcomes that can be of help to patients and clinicians for the management of their care. Another limitation is that not all currently available biomarkers were included in this analysis, such as the presence of IgG or IgM oligoclonal bands, neurofilaments light chain or chitinase-3 from CSF samples, which may have contributed to improving the accuracy of the prognosis algorithms.

In summary, we found that machine learning algorithms for predicting relevant clinical outcomes in the short term for MS patients achieve intermediate to good accuracy using data that is commonly collected at the outpatient clinic, such as disability scales or imaging. Although omics improved the accuracy slightly in some cases, at present, the information they provide is not worth the cost and efforts they will imply. Future studies with more informative biomarkers might improve the accuracy for predicting disease course.

### Supplementary Information

Below is the link to the electronic supplementary material.

Supplementary file1 (XLSX 60 KB)

## Data Availability

Sequence data have been deposited at the European Genome-phenome Archive (EGA), under accession number EGAS00001007145 (https://ega-archive.org/studies/EGAS00001007145). The code with the trained random forest algorithms is available at https://github.com/anafreire/sys4ms

## Declarations

### Statistical analysis

The machine learning analysis was conducted by Magi Andorra, Ana Freire, and Ricardo Baeza-Yates, and supervised by Pablo Villoslada.
